# Fabrication of Silver-Decorated Graphene Oxide Nanohybrids via Pulsed Laser Ablation with Excellent Antimicrobial and Optical Limiting Performance

**DOI:** 10.3390/nano11040880

**Published:** 2021-03-30

**Authors:** Parvathy Nancy, Jiya Jose, Nithin Joy, Sivakumaran Valluvadasan, Reji Philip, Rodolphe Antoine, Sabu Thomas, Nandakumar Kalarikkal

**Affiliations:** 1 School of Pure and Applied Physics, Mahatma Gandhi University, Kottayam 686560, India; parvathy88.nancy@gmail.com; 2 International and Inter University Centre for Nanoscience and Nanotechnology, Mahatma Gandhi University, Kottayam 686560, India; jiyajose@gmail.com (J.J.); sabuthomas@mgu.ac.in (S.T.); 3 Light & Matter Physics Group, Raman Research Institute, Bengaluru 560080, India; nithinpglm@gmail.com (N.J.); philipreji@gmail.com (R.P.); 4 Accelerator Division, Institute of Plasma Research, Near Indira Bridge, Gandhinagar District, Bhat, Gujarat 382428, India; siva@ipr.res.in; 5 CNRS Institut Lumière, Matière Université Claude Bernard, Univ Lyon, Lyon 1, F-69622 Lyon, France; 6 School of Chemical Sciences, Mahatma Gandhi University, Kottayam 686560, India

**Keywords:** pulsed laser ablation, silver nanoparticles, graphene oxide, Ag-GO nanohybrid material, antimicrobial activity, optical limiting

## Abstract

The demand for metallic nanoparticle ornamented nanohybrid materials of graphene oxide (GO) finds copious recognition by virtue of its advanced high-tech applications. Far apart from the long-established synthesis protocols, a novel laser-induced generation of silver nanoparticles (Ag NPs) that are anchored onto the GO layers by a single-step green method named pulsed laser ablation has been exemplified in this work. The second and third harmonic wavelengths (532 nm and 355 nm) of an Nd:YAG pulsed laser is used for the production of Ag NPs from a bulk solid silver target ablated in an aqueous solution of GO to fabricate colloidal Ag-GO nanohybrid materials. UV-Vis absorption spectroscopy, Raman spectroscopy, and TEM validate the optical, structural, and morphological features of the hybrid nanomaterials. The results revealed that the laser-assisted in-situ deposition of Ag NPs on the few-layered GO surface improved its antibacterial properties, in which the hybrid nanostructure synthesized at a longer wavelength exhibited higher antibacterial action resistance to *Escherichia coli* (*E. coli*) than *Staphylococcus aureus* (*S. aureus*) bacteria. Moreover, nonlinear optical absorption (NLA) of Ag-GO nanohybrid was measured using the open aperture Z-scan technique. The Z-scan results signify the NLA properties of the Ag-GO hybrid material and have a large decline in transmittance of more than 60%, which can be employed as a promising optical limiting (OL) material.

## 1. Introduction

Graphene, a one-atom-thick carbon derivative in which a few layers of carbon atoms arranged in a hexagonal 2D lattice, is recognized as an exemplary matrix for the immobilization of NPs, due to its superior thermal/chemical stability, large surface area, and strong coupling between various nanoparticles and graphene for a large number of photonic and electronic applications [1,2,3]. In addition, because of the existence of epoxy (–COO−) and hydroxyl (OH−) groups about the basal plane and carboxyl groups (–COO−) near the edge of the molecular structure, graphene oxide (GO) can interact with numerous organic/inorganic materials [4,5,6]. GO has also been recommended for a plethora of biomedical usages comprising disinfection applications [7]. The antibacterial efficacy of GO can be enhanced by the inclusion of silver nanoparticles (Ag NPs) towards both gram-negative and gram-positive bacteria, with multifaceted destruction activity. Furthermore, GO exhibits remarkable nonlinear optical (NLO) effects such as enhanced second-harmonic generation (SHG), two-photon absorption (TPA), and reverse saturable absorption (RSA) compared to the pristine graphene [8,9,10]. Additionally, GO, when covalently functionalized with zinc phthalocyanine, exhibits remarkable nonlinear absorption effects over a broad spectral region specifying its great ability to be applied in micro-photonic devices such as optical limiter and optical switcher [11]. Indeed, molecules, clusters, and nanoparticles can be efficiently captured due to the large surface areas of graphenic materials. The coalition of the two classes of materials (graphene and nanoparticles) may give rise to the integration of properties of each component in a new hybrid material that holds important features for a variety of application interests. Plasmonic metallic nanomaterials (Ag, Cu, and Au) with enough rough surfaces could provide unprecedented strong and intense electromagnetic fields because of their rugged structures. Thus the graphene-nanoparticle hybrid materials are particularly relevant because they exhibit the individual properties of the nanoparticles and graphene as well as additional synergistic properties, thereby achieving sensitivity and selectivity towards a variety of sensing applications. The main hindrance to the overgrowth/deposition of NPs on graphene layers is the lack of strong and facile generation strategies, which is a real challenge in the present nanofabrication technology owing to the very small dimensions. Basically, two main synthesis approaches have been proposed to produce graphene-nanoparticle composites: in-situ and ex-situ routes [12]. For instance, we recently used ex-situ methods to covalently anchor atomically precise glutathione-protected gold nanoclusters onto graphene oxide, displaying enhanced NLO signals [13]. However, the main advantage of in-situ synthesis is the simplicity to obtain high surface coverage NPs decorated on GO surfaces, as demonstrated by us with gold NPs [14]. The existing and widely used in-situ techniques such as chemical (chemical reduction, photochemical reaction, electrolyte plating, chemical functionalization, electrostatic self-assembly, etc.) and physical vapor deposition (thermal evaporation, pulsed laser deposition) processes are formulated on a bottom-up nanofabrication approach and having so many demerits such as usage of various additives and less control on the size, density, and composition of the NPs generated [15,16,17,18,19,20,21,22,23,24,25,26,27]. In addition, because of the difficulties to coalesce the huge-sized NP and graphene, the composites/hybrid materials synthesized so far constitute macroscopic powder aggregates and irregularities, which is fruitless for some applications such as surface-enhanced Raman spectroscopy (SERS). Herein, this study is a novel way to overcome such impediments through laser ablation in ambient liquid. Pulsed laser ablation in liquid medium is a one-pot, top-down synthesis strategy and is a sophisticated and versatile synthesis tool with wide acceptance in generating highly pure NPs [28,29]. This in-situ technique has advantages such as clean and control of particle dimensions in the range of a few to several hundreds of nanometers. Furthermore, one can perform comparatively better controlled, safe, and in-situ synthesis of metallic nanoparticles, which does not require the post-production purification process as there are no impurities or unwanted objects produced during the ablation [30,31,32,33]. The laser parameters, target material, and the liquid medium, which imparts compact confinement of the ablated material, have a key role in the properties of the final product. The presence of the materials such as graphene sheets in the ablation medium may cause the generation of unique structures. The authenticity and importance of the proposed synthesis route is manifested by an interesting report describing the synthesis of ligand-free Au atom clusters adsorbed on graphene sheets by oxidative laser ablation and fragmentation in water by M Lau et al. [34]. Moreover, there are so many thought-provoking reports such as novel laser-based in-situ bio-conjugations for drug delivery/bio-sensing/diagnostics applications, fabrication of bio-functional nanoprobes such as *S. aureus* protein-capped silicon quantum dots based on infrared ultrafast laser ablation, etc. [35,36].

The microbial resistance to various antibiotics/biocides is a crucial universal menace to public health parallel to that caused by global warming and terrorism as per the reports published by the World Health Organization (WHO) [37]. Graphene-based nanomaterials have emerged as a favorable medium for antibacterial activity in recent years [38,39]. The exposure with such materials having various exogenous functional groups, the membrane stress produced by the sharp-edged graphene nanosheets has resulted in remarkable physical destruction to the cell membrane, leakage of intracellular material, and successive loss of membrane integrity of the bacteria [40]. The antimicrobial effects of silver and silver-decorated nanomaterials are well-studied for the past three decades [41,42,43,44]. An effective strategy to improve the antibacterial activity can probably be accomplished by integrating GO with Ag NPs. The present study explores the possibilities to enhance the oxidative and membranolytic activity of GO with the laser-assisted decoration of Ag NPs. This investigation aims to check the activity of Ag-GO nanohybrids fabricated via laser ablation against infectious multi-drug resistant pathogens, such as *Escherichia coli* (gram-negative) and *Staphylococcus aureus* (gram-positive). Metal nanoparticles wrapped in two-dimensional materials such as graphene oxide (GO) have captivated much attention due to their excellent linear and nonlinear optical (NLO) features [45,46,47]. Since it was first studied by Wang et al., so many related studies have been performed to realize the optical limiting response and the fundamental processes of carbonaceous materials, such as graphene, graphene oxide (GO), and composites of graphene [48,49,50]. NLO responses of silver incorporated graphene in picoseconds regime by Z-scan technique under the excitation wavelength, 1064 nm was investigated by Kalanoor et al. [51]. Sadrolhosseini et al. have studied the NLO features of the Au-GO nanocomposites, synthesized by pulsed laser ablation technique [52]. Yue et al. and Li et al. reported the improved nonlinear optical properties of silver-decorated graphene using reduced graphene oxide as a 2D template [53,54]. Instead of using planar graphene nanosheets, the ridged/wrinkled graphene (GO, rGO) has so many advantages in binding various metal nanoparticles and may also contribute to enhancing NLO response [8,25]. The NLO properties of laser-synthesized Ag-GO hybrid nanocolloids have not been studied thoroughly by Z-scan in a nanosecond time scale. (i.e., at the near-resonant excitation wavelength, 532 nm). Hence, this work also aims to gather insights into the nonlinear optical properties of Ag-GO nanohybrids examined particularly in the nanosecond regime by the Z-scan method as a function of the input fluence of the incident radiation.

Here in this work, we report the antibacterial and NLO properties of nontoxic ligand-free Ag-GO nanohybrids with unique properties such as absolute purity, narrow and homogeneous particle size distribution, and electrostatic stability, fabricated via pulsed laser ablation for the first time. Compared to the seminal/previous works in this domain, our study reflects better insights towards the synthesis and application potentialities of the Ag-GO hybrid nanocolloids with precise size control in less reaction time by tuning the laser parameters [55,56,57].

## 2. Experimental Details

The experiment has been established in two different steps.

(1) Synthesis of GO, using the modified Hummers method [3]. The synthesis protocol is explained as follows:

Natural graphite powder was incorporated for the synthesis of GO. A total of 1 g of sodium nitrate and 3 g of natural graphite were added to sulfuric acid (46 mL) and maintained in an ice bath with stirring. Then, 6 g of potassium permanganate was moderately added to this composition, and the solution was preserved below 20 °C with stirring. After that, the solution temperature was raised to 35 °C, and the solution was again stirred for one hour. Then, deionized water (40 mL) was slowly added to this solution, stirred for 30 min, and again diluted with deionized water (100 mL) in an ice bath to avert quick boiling because this procedure gives rise to a sudden increase in temperature. Finally, hydrogen peroxide (6 mL, 30%) was slowly added to the mixture. This resulted in the color change of the solution to bright yellow along with bubble formation. The final solution was filtered through filter paper (Standard Whatman filter paper having pore size 11 µm) and washed with deionized water until the filtrate was neutralized. The filtered material was then dried in a vacuum oven for 48 h. For attaining the proper vacuum conditions, the pressure was set to 10^−3^ mbar. The as-prepared GO nanosheets were used as the matrix for the in-situ decoration of Ag NPs via PLAL.

(2) The synthesis of Ag-GO nanohybrids by ablating an Ag target in the aqueous suspension of GO nanosheets.

**Figure 1:**
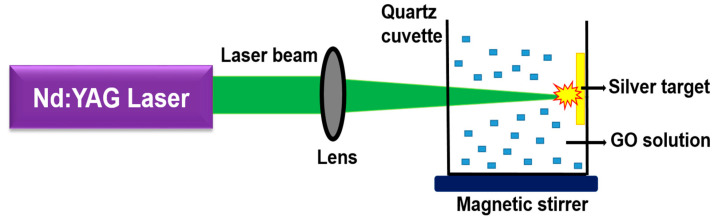
The experimental setup of production of colloidal silver graphene oxide (Ag-GO) nanohybrid.

The colloidal Ag-GO nanohybrid materials were synthesized by pulsed laser ablation of a metal target (Ag) in the aqueous solution of GO. The GO concentration of the solution was approximately 0.5 mg mL^−1^. Figure 1 represents the experimental layout of pulsed laser ablation. A 1 mm thick solid Ag target (pure trace metal, SIGMA ALDRICH, 99.99%) was properly fixed in the inner sidewalls of a quartz cuvette, which contained 30 mL of GO solution. A Q-switched pulsed Nd-YAG Laser (Litron LPY 674G-10) beam with 8 ns pulse width and 10 Hz repetition rate was allowed to focus on the Ag target with the aid of a biconvex lens having focal length (15 cm). The whole experiment was conducted at room temperature. The Ag target was ablated at 400 mJ of laser energy in two different laser wavelengths (532 nm and 355 nm). The GO solution was kept under stirring continuously during ablation by employing a magnetic stirrer. The ablation time was initially set to be 15 min for the generation of Ag-GO hybrid nanocolloids. Moreover, the experiment was performed for different ablation times, such as 18 min, 21 min, 24 min, and 27 min.

## 3. Results and Discussion

PLAL has been manifested to be a robust synthesis method for nanoparticle generation [58]. This procedure requires so many tunable parameters, which should be precisely optimized. In this study, liquid-phase laser ablation of an Ag target in GO was performed at two different wavelengths in a nanosecond regime. Successive to the laser ablation, Ag NPs are firmly bounded on the surface of each graphene layer, resulting in a hierarchical colloidal nanohybrid. The concentration and size of these NPs can be controlled by the various parameters of the laser. The pulsed laser ablation of a bulk Ag target in a liquid medium is a highly clean process that guarantees high surface activity of Ag NPs. The cleanliness of the pulsed laser ablation cannot be attained by even the green chemistry methods where there are lots of possibilities for the existence of metal ions in their unreduced form in the final product. Hence, this technique can be used to embellish GO nanosheets with different NPs as per the requirement than Ag, and that can also be fabricated from the simple metals/hybrids and semiconductors. The as-prepared Ag-GO nanohybrids have improved bactericidal effects to design and develop antibiotics based on graphene for biomedical applications and optical limiting performance against hazardous laser beams. Employing the unique properties of colloidal state Ag-GO nanohybrid material has promising applications in making so many nano-devices and sensors.

### 3.1. UV-Visible Absorption Spectroscopy

The in-situ generation of Ag NPs over GO sheets is confirmed by absorption spectroscopy. Each UV absorption spectra have been recorded in a range of 200−800 nm. All the optical absorption spectra of the colloidal Ag-GO nanohybrids synthesized at various experimental conditions have been compared with that of the bare GO colloids.

**Figure 2:**
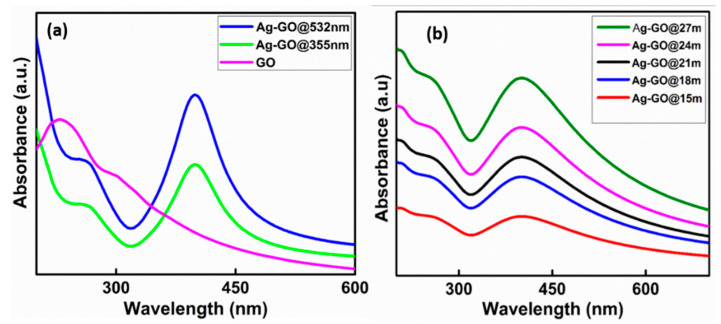
UV-Vis absorption spectra of (**a**) bare GO and laser-synthesized Ag-GO nanohybrid samples at 355 nm and 532 nm laser wavelengths and (**b**) Ag-GO nanohybrids synthesized at different ablation times (15, 18, 21, 24, and 27 min).

Figure 2a illustrates the UV-Vis absorption spectrum of bare GO nanosheets and Ag-GO nanohybrids synthesized at second (532 nm) and third (355 nm) harmonic wavelengths. The bare GO nanosheets reveal an absorption peak at 234 nm, which is attributed to π → π* interaction of C-C bonds and a tiny shoulder peak at 304 nm corresponding to n → π* interaction of C = O bonds. After the laser ablation, the GO absorption peak slowly shifts to 255 nm, which designates that a few GO layers have been reduced because of the interaction of GO with high laser energy. Moreover, the whole Ag-GO sample reveals an absorption peak at ~402 nm, which is attributed to the surface plasmon resonance (SPR) of Ag NPs [59,60]. It implies that the Ag NPs have been generated and well-anchored on the surface of GO nanosheets. If the GO colloidal concentration, ablation wavelength (532 nm), and laser energy (400 mJ) are fixed, the density of the Ag NPs on the GO nanosheets has been increased with the increase of ablation time (Figure 2b) [61]. A high rate of Ag NPs production is observed at a larger laser wavelength and higher ablation time. The higher absorbance at a longer wavelength is due to the increase in particle concentration. Especially in the case of silver, strong absorption is because of surface plasmon resonance in the range of the second-harmonic wavelength (532 nm) of the laser. In the case of a longer wavelength (532 nm), there exists a possibility that each wave can interact more with the surface of the bulk silver target. Hence, the corresponding electromagnetic interactions are more in this case compared to short wavelengths (355 nm) so that the nanoparticle yield is high at 532 nm. That is why the optical density is higher in the case of 532 nm synthesized Ag-GO hybrids (Figure 2a). The dynamics of plasma plume in a liquid environment, laser parameters, nonlinear absorption in the liquid, particle fragmentation, etc., are the other key controlling parameters of the nanoparticle production rate [62,63,64,65].

In agreement with the generally accepted growth mechanism of NPs, it is inferred that a condensed cloud of Ag clusters and atoms formed on the target surface, particularly at the vicinity of the laser beam spot, instantly just after the ablation process [31]. Thereafter, the ablated species bring together/clump into minute embryonic NPs rapidly and grow until every atom nearby the embryonic NPs is consumed completely. In this present case, the liquid medium contains GO sheets, and the Ag NPs straightaway sintered with GO nanosheets. Such a phenomenon is identical to the nano-soldering, which is used to describe the nano-webs comprised of Pt NPs soldered by molten Au while laser ablation of a mixed solution of Au and Pt NPs [66]. For now, the Ag NPs that are not being anchored on the surface of GO layers experience Brownian motion because of their small dimensions. Natural collisions can occur between the free-moving Ag NPs in the colloidal solution, and the GO nanosheets with micro-sized slanting dimensions result in their successive sintering by absorbing the energy of photons.

### 3.2. Raman Spectroscopy

**Figure 3:**
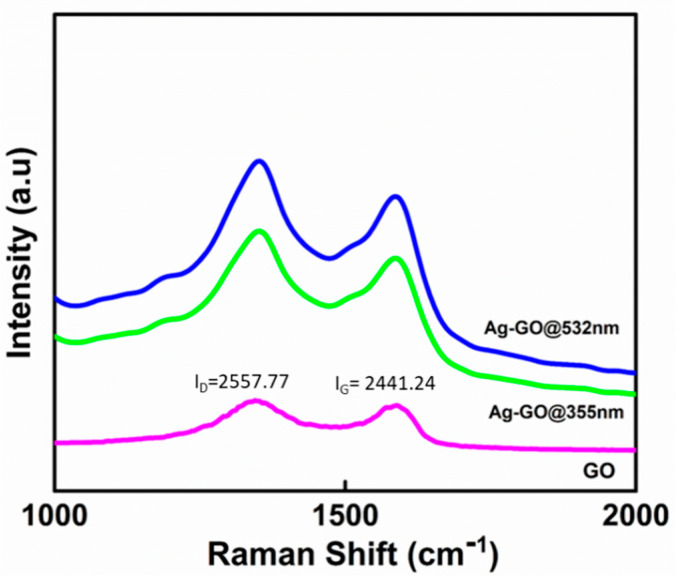
Raman spectra of the GO and Ag-GO nanohybrids.

Raman spectroscopy studies were carried out to understand additional information on the chemical fingerprint of the Ag-GO nanohybrids (Figure 3). The background was eliminated as per this reference [67].

The Raman spectra show an intense peak at the D band (1350 cm^−1^) and a G band peak at 1589 cm^−1^, which declares the graphitic structure of a few-layered GO. The I_D_/I_G_ ratio/value is used as a measure of the disorder/restoration of the graphene lattice, standing for the sp^2^/sp^3^ carbon ratio. The increased I_D_/I_G_ value (1.14) of Ag-GO@532 nm is higher than that of both Ag-GO@355 nm (1.12) and bare GO (1.04), indicating that the Ag-GO@532 nm is having a higher concentration of Ag NPs; therefore, extra defects were introduced to the graphene sheets. These defects are allotted to the graphitic oxidation and Ag decoration effects in the hexagonal carbon lattice. Furthermore, it is noted that Ag-GO@532 nm and Ag-GO@355 nm exhibit higher Raman intensities than that of bare GO. This is because of the enhancement in the local electromagnetic field, which again substantiates the successful generation and anchoring of Ag NPs on the surface of GO layers. The intensified Raman signals of the Ag-GO samples compared with the bare GO is because of the effect of surface-enhanced Raman scattering due to the presence of Ag NPs [68]. Evidently, the generation of Ag NP during pulsed laser ablation and the interfacial binding between GO and Ag does not seriously affect the properties of the Ag NPs. Hence, compared to other chemical reduction methods, the pulsed laser ablation extends the advantage of contamination-free and a tunable decoration of Ag NPs on a few-layered GO, which is fundamentally important for various prominent surface activities.

### 3.3. Morphological Analysis

Direct quantification of the size of the Ag NPs and the evidence for the formation of Ag-GO nanohybrids has been made possible through the transmission electron microscopy (TEM) and high resolution transmission electron microscopy (HRTEM) images.

**Figure 4:**
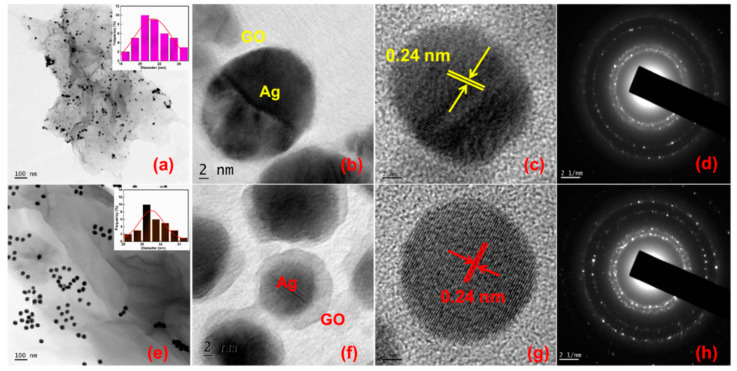
TEM and HRTEM images of (**a**–**c**) Ag-GO@355 nm, (**e**–**g**) Ag-GO@532 nm samples synthesized at 15 min ablation time and (**d**,**h**) selected area electron diffraction (SAED) patterns of Ag-GO@355 nm and Ag-GO@532 nm samples, respectively.

The TEM results indicate that the individual GO sheets are heavily coated with Ag NPs revealing a hierarchical hybrid nanostructure. Figure 4a,e depicts the TEM images of Ag-GO@355 nm and Ag-GO@532 nm synthesized at 15 min ablation time. Figure 4b,c,f,g shows the HRTEM images of the intersection between Ag NPs and transparent and wrinkled GO layers and the lattice fringes of laser-generated Ag-GO@355 nm and Ag-GO@532 nm nanohybrids, respectively. The d-spacing value, 0.24 nm, which represents the (111) plane of the face-centered cubic (FCC) Ag NPs, is clearly observed from the HRTEM image [59]. Figure 4d,h shows the selected area electron diffraction (SAED) pattern, which reveals the characteristic diffraction spots arising from the laser-produced Ag-GO@355 nm and Ag-GO@532 nm nanohybrids exhibit high crystallinity. The Ag NPs are almost spherical in shape with an average particle size of 20 ± 4 nm for Ag-GO@355 nm and 30 ± 5 nm for Ag-GO@532 nm, respectively. The inset of Figure 4a,e represents the particle size distribution illustration. The number of particles investigated for constructing the histogram was 40. The particle size is determined using ImageJ software. The as-synthesized hybrid nanostructures, aggregation of both Ag NPs and the GO layers, are effectively less because of the fact that the GO layers serve as a planar substrate to affix the 0D Ag NPs, while these metal NPs serve as spacers between the GO nanosheets. There are some electrostatic interactions between the Ag cations with the oxygen functional groups in GO during the laser-induced in-situ decoration of Ag NPs on the GO surface. The negatively charged GO sheets permit the smooth anchoring of positively charged metal (Ag) ions. The size of the Ag NPs can be tuned by changing the photon energy of the laser (wavelength). The impact of laser wavelength on Ag NP size can be explained in terms of ablation efficiency, self-absorption, penetration depth of laser beam, etc. [69]. The result shows that the average particle size is decreased for the sample (Ag-GO@355 nm) prepared at shorter wavelength. In the present work, no harmful stabilizing, dispersing, and reducing agents were used to prepare the Ag-GO nanohybrids, as long as most of the previously reported literature uses so many hazardous chemicals. Thus this work explores an eco-friendly synthesis approach that fits the principles of green chemistry and a great advantage of the elimination of unwanted residues/reducing agents and chemical ligands for the fabrication of various hybrid nanomaterials for multifunctional applications.

### 3.4. Antibacterial Activity

In order to explore the possibilities of the as-prepared Ag-GO nanohybrids in the biomedical field, the antimicrobial activity of GO, Ag NPs, Ag-GO@355 nm, and Ag-GO@532 nm was examined against *Escherichia coli* (*E. coli*) and *Staphylococcus aureus* (*S. aureus)*. The antimicrobial activity was investigated using the Muller–Hinton agar well diffusion method and was prepared in agar plates with 24 h old cultures [70]. The microorganisms were uniformly swabbed on nutrient agar-agar plates using a sterile cotton swab, and then 6 mm diameter wells were created in the corresponding plates by employing a sterile well borer. The samples were poured into each well with the aid of a micropipette. The amount of sample poured was 100 µL. The plates were incubated at 37 °C for 24 h for the microorganisms to grow, and after that, the diameter of the inhibition zones has been measured and are tabulated in Table 1 and Table 2.

**Figure 5:**
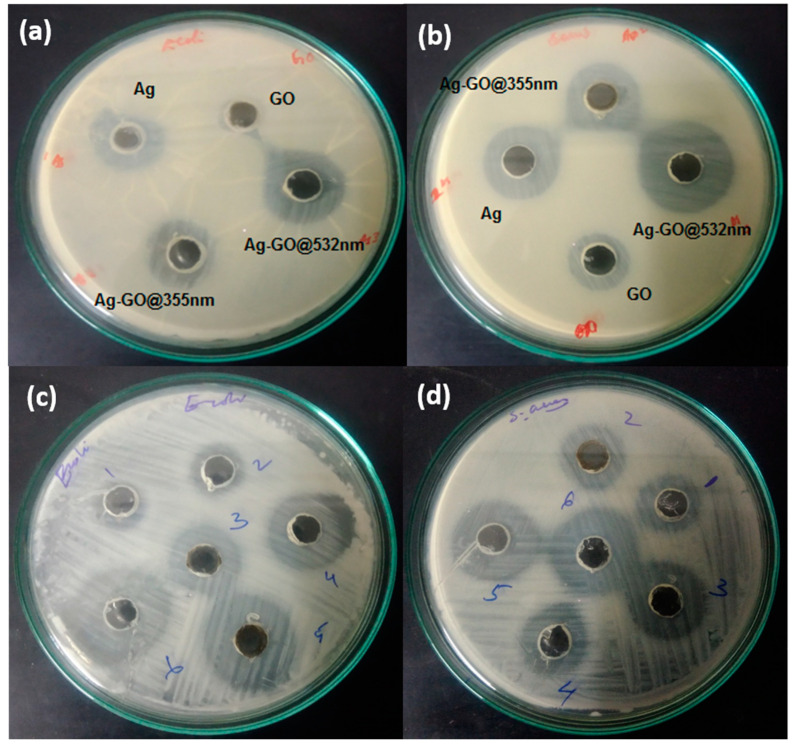
Inhibition zone images of (**a**) *E. coli* for GO, silver nanoparticles (Ag NPs), Ag-GO@355 nm and Ag-GO@532 nm, (**b**) *S. aureus* for GO, Ag NPs, Ag-GO@355 nm and Ag-GO@532 nm, (**c**) *E. coli* for Ag-GO@532 nm synthesized at five different ablation times, and (**d**) *S. aureus* for Ag-GO@532 nm synthesized at five different ablation times.

It is well understood from the images that both gram-negative and gram-positive strains were susceptible to the Ag-GO nanohybrid. The Ag-GO nanohybrid was synthesized at two different laser wavelengths and at five different time scales by keeping the laser energy constant at 400 mJ. The visible clear inhibition zones generated by the activity of Ag NPs, GO, Ag-GO@355 nm and Ag-GO@532 nm against the *E. coli* and *S. aureus* gives a clear evidence of the antibacterial efficiency. From Figure 5a,b, it is inferred that the zone diameter for the Ag-GO nanohybrid synthesized at 532 nm is larger than the others. Moreover, when the ablation time is increased from 15 to 27 min, the inhibition zone diameter became larger; from this, it can be concluded that the Ag NPs concentration was increased at higher ablation time. The same trend has been observed in the case of both gram-positive and gram-negative bacteria. Figure 5c,d illustrates the images of inhibition zone diameters of *E. coli* and *S. aureus* treated with Ag-GO@532 nm prepared at five different ablation times. Hence the activity is found to be higher for samples synthesized at larger wavelength and time frame.

**Figure 6:**
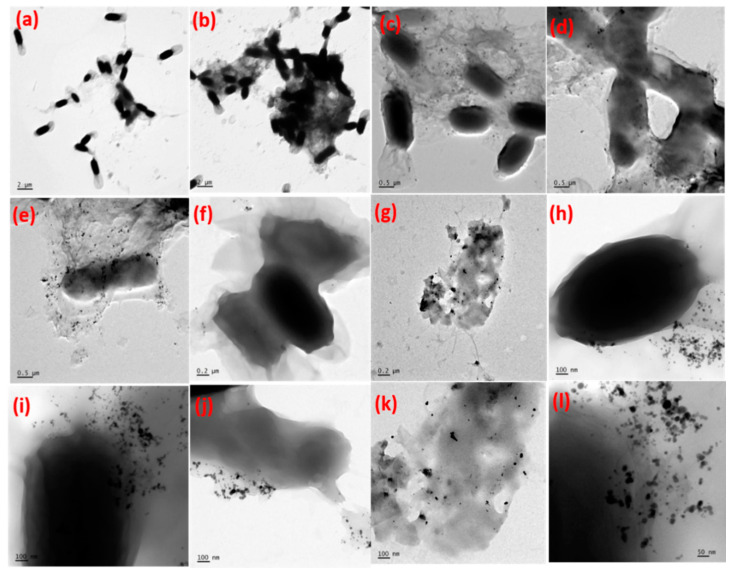
The TEM images of (**a**,**b**) *E. coli* bacteria treated with the sample Ag-GO@532 nm synthesized at the ablation time 15 min and (**c**–**l**) various destruction stages of *E. coli* bacteria.

Added evidence to the results, *E. coli* treated with the nanohybrid materials has been analyzed under TEM to understand the mode of action towards the bacterial culture. Ag NPs are known for their antibacterial activity, but the real mechanism of action is not well understood. Since the antibacterial activity of Ag-GO@532 nm samples is found to be more effective than the Ag-GO@355 nm samples, the activity of Ag-GO@532 nm samples toward gram-negative bacteria (*E. coli)* has been further confirmed by the TEM observations and is illustrated in Figure 6. The GO layers easily absorb and embed on the surface layers of the bacterial cells. It can induce oxidative stress to the bacterial cell walls. The chemical composition and rough/sharp edges of the graphene sheets are capable of piercing the cell membrane. Ag NPs have the ability to penetrate into the cell wall of both gram-negative and gram-positive bacteria. The cell walls of the bacteria were damaged, and further cell multiplication was stopped by the action of silver NPs. From the results, it is inferred that the Ag-GO@532 nm samples were well penetrated into the cell walls, and the whole cells were disrupted.

The above outcomes suggest that the laser-induced in-situ decoration of Ag NPs on the GO nanosheets upgraded its antibacterial property against bacterial species such as *E. coli* and *S. aureus*. It is reported that the Ag-GO nanohybrid exhibits more antibacterial to gram-negative (*E. coli*) bacteria than gram-positive (*S. aureus*) strains. The effect is attributed to the structural dissimilarities in the cell membranes of gram-positive and gram-negative bacterial strains [71]. The peptidoglycan layer in the cell walls of gram-negative (1–2 layers) bacteria is generally thinner than that of the gram-positive (10 layers) bacteria [72]. Thus, the thinner cell walls of *E. coli* can significantly facilitate the transmembrane movement of toxins from the solution, which promotes a higher antibacterial activity. A similar result was reported earlier by Shao et al. in bacterial strains. The mechanism involved was the connection of Ag NPs with the bacterial cell surface, which is assumed to be mediated by the electrostatic interplay between the positively charged nanoparticles and negatively charged cell membrane of the bacteria [73,74]. As soon as the Ag NPs adhere to the microbial cells, they can easily pierce the cell walls, leading to the destruction of their internal constituents. The cellular incorporation of Ag NPs is also capable of initiating the generation of reactive oxygen species (ROS) and activating oxidative stress in the microbial cells due to Ag^+^ ion discharge. It is reported that the oxidation of the surface of Ag NPs in the presence of oxygen dissolved in the aqueous suspension and Ag^+^ ions is created by the oxidative dissolution of Ag NPs [75]. As a result, both Ag NPs and Ag^+^ ions can damage bacterial cells and can be the cause of the deactivation of proteins in the bacterial cell wall, which eventually leads to the death of the microorganisms. Based on this, the antibacterial activity of Ag-GO samples could be ascribed to the synergistic effect of GO and the silver ion dissolution from the Ag NPs to the microbial cells. The results of the antibacterial activities revealed that the Ag-GO nanohybrids have more improved antibacterial properties than Ag NPs and GO, in which higher antibacterial activity is against *Escherichia coli* (*E. coli*) than *Staphylococcus aureus* (*S. aureus*) bacteria.

### 3.5. Nonlinear Optical Studies

To probe the NLO performance of the synthesized samples to function as a good optical limiting (OL) material under nanosecond excitation regime (532 nm) and to understand the responsible mechanism for the optical nonlinear behavior, Z-scan studies of the Ag NPs, GO, and Ag-GO nanohybrids were carried out. When the intensity of the laser light is adequately strong, then the probability of absorbing more than a single photon in an event increases, and that results in optical nonlinearity. So many nonparametric phenomena occurred because of the existence of a real excited state, which is attainable through excited state and multiphoton absorption within the time span of the incoming laser beam. Basically, these kinds of phenomena happen through various interesting processes such as multiphoton absorption (MPA), free carrier absorption (FCA), and excited-state absorption (ESA), contingent on intensity, excitation wavelength, and laser pulse width.

**Figure 7:**
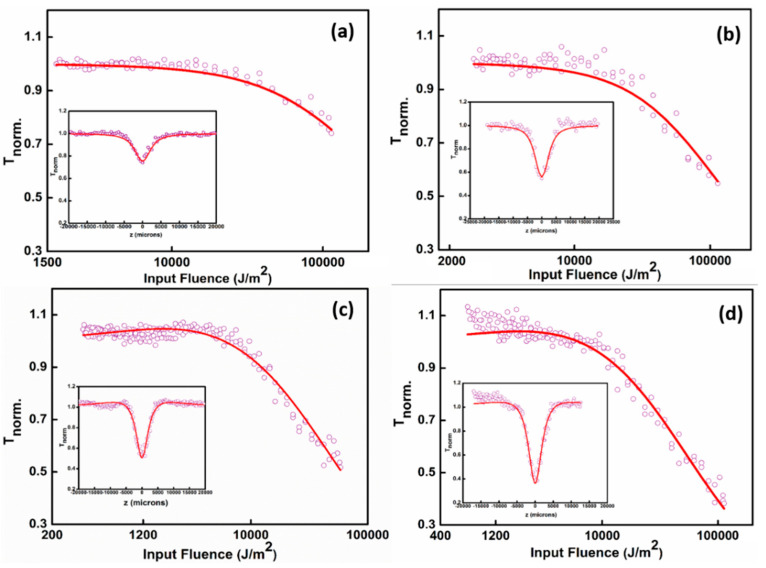
Optical limiting curves of (**a**) Ag NPs, (**b**) GO, (**c**) Ag-GO@355 nm, and (**d**) Ag-GO@532 nm synthesized at the ablation time of 15 min. Laser pulse excitation at a laser pulse energy of 40 µJ. Open circles: data points, solid lines: numerical fits to the corresponding data points.

The nonlinear optical properties (NLO) of the Ag NPs, GO, and Ag-GO nanohybrids have been examined by the Z-scan technique equipped with a Q-switched pulsed Nd:YAG laser (Minilite Continuum), which delivers a second-harmonic Gaussian beam at a wavelength of 532 nm at a repetition rate 10 Hz with a pulse duration of 5 ns. The laser beam is focused on the colloidal samples through a plano-convex lens having a focal length = 10.75 cm. The samples are taken in a 1 mm path length cuvette. The cuvette is mounted on a linear translational stage controlled by a stepper motor and is translated along the *z*-axis through the focal position. The sample experiences different laser fluences in every z position, and the transmittance is detected by a pyroelectric energy probe (RjP-735, Laser Probe Inc., New York, USA) placed behind the sample. In all measurements, the laser output is externally triggered and is running in a single-shot mode. A noticeable improvement in the NLO features of Ag-GO nanohybrids has been reported in comparison with those of the bare GO nanosheets and Ag NPs. The increased NLO response in Ag-GO nanohybrids may be ascribed to the complex energy band structures developed during the synthesis, which supports resonant transitions to the conduction band through surface plasmon resonance (SPR) at lower laser intensities and ESA to the GO conduction band at higher laser intensities [55]. The photo-generated charge carriers in the Ag conduction band or the hike in defects during the generation of Ag-GO nanohybrids may promote ESA. Figure 7 shows the open aperture Z-scan results of the samples. The insets show the Z-scan curves, which explain the normalized transmittance of the samples given as a function of the position of the samples. Open aperture Z-scan results indicate reverse saturable absorption (RSA) behavior of the prepared Ag-GO nanohybrids, which is a clear manifestation of its optical limiting ability. The values of nonlinear absorption coefficients have been extracted by numerically fitting the experimental data using nonlinear transmission equations [76].

Generally, the nanosecond Z-scan experiment results show a strong dependence of the nonlinear absorption coefficient (β_eff_) with input laser fluence [76]. Usually, in carbon materials, the optical limiting performance of nanosecond laser pulses is mostly created by ESA. Pure graphene dispersions exhibit significant NLO properties ascribed to saturable absorption (SA) at low input energy and two-photon absorption (TPA) at high incident intensities. In addition, Ag NPs have shown NLO ability, which corresponds to ESA related to FCA in the nanosecond regime. From these states, the collective effect of all the above-stated NLO mechanisms (TPA, ESA, and FCA) promotes the conception of these novel hybrid materials with distinctive NLO features [77].

The optical nonlinearity of the Ag-GO@532 nm sample is far higher compared to bare GO, Ag NPs, and Ag-GO@355 nm, which is illustrated in Figure 7a–d. By exciting the samples with the laser radiation, the electrons will transfer to the graphenic carbon excited state; from there, it can effortlessly go to the unoccupied d orbitals of Ag NPs. There is also a possibility of electron transfer from the excited state to the Ag NPs valance band. After that, there exists a chance of electronic transition to higher excited states via absorbing another photon. Additional absorption of a photon leads the electron to a continuum through FCA. From Figure 7, it is evident that the Ag-GO colloidal hybrid nanomaterials exhibit good OL response than the bare GO and Ag NPs alone. From the Z-scan results, it can be clearly inferred that the Ag-GO samples synthesized at longer wavelengths (532 nm) are highly preferred to have improved OL properties than that of the hybrid materials generated at shorter laser wavelengths. This is due to the fact that the Ag-GO synthesized at 532 nm constitute bigger and denser Ag NPs so that the surface area is large, which is favorable for the strong nonlinear absorption (NLA). The Ag-GO@ 532 nm has a higher β*_eff_* value compared to the other samples, which confirms the better OL properties of the hybrid material. Furthermore, the interaction between the energy states of Ag NPs and graphene sheets in Ag-GO nanohybrids will activate the creation of complex energy states, which can be evidenced from the increased intensity in the Raman spectra of the hybrid structures, and hence the corresponding enhancement could be ascribed to SPR of Ag NPs anchored on the GO layers and charge/energy transfer. Here in the case of Ag-GO nanohybrid materials, it is significant to note that the value of β*_eff_* is highly governed by ESA and FCA, and relatively less contribution is from the direct TPA. The higher contribution by ESA arises from the presence of intermediate energy levels, which results in a large reduction in population in the ground level energy state. The population of these intermediate levels becomes dense, and further photon absorption from this level can occur under enough powerful input laser intensity. The availability of an immense number of free electrons in the metallic Ag NP system enables energy absorption via Ohmic conduction, which is the reason for the enhancement in NLA in the Ag-GO hybrid nanomaterials [78]. Here, the synergistic relationship between the metallic Ag NPs and defective graphene layers improves the NLA in Ag-GO samples compared to the bare Ag NPs and GO. The obtained nonlinear parameters for laser pulse excitations in the nanosecond regime are enlisted below in Table 3. In Figure 7d, at low input fluence, a slight deviation is observed for the TPA fit. This deviation can be attributed to the variation in the Gaussian profile of the laser pulse during the scanning process, in particular at low laser fluence. The laser beams at 532 nm and 355 nm are obtained with second and third harmonics generation of the fundamental laser beam, and thus the laser beam profiles are supposed to be different. Of note, this process inducing the third photon is more efficient at 532 nm. Of note, it appears that deviations occur when *β_eff_* are very high (Ag-GO@532 nm), where higher-order effects (e.g., three-photon) may occur. This effect has been already observed for other nanostructured materials [79,80] and leads to a similar deviation in the low laser fluence part of the Z-scan curves. Of note, this process inducing the third photon is more efficient at 532 nm (close to the plasmon resonance) than at 355 nm (more in the intra-band excitations).

The numerically calculated values of nonlinear optical parameters of the samples produce better insights toward the OL features of the Ag-GO nanohybrids synthesized via pulsed laser ablation, particularly in the nanosecond regime in a very short span time (15 min ablation time). Compared to the previous reports, the NLO responses of laser-induced colloidal Ag-GO nanohybrid materials in nanosecond regime are less investigated, and the present work establishes the wavelength and ablation time-dependent in-situ growth of metal NPs ornamented 2D hybrid materials for the first time [50,52,81]. The nonlinear optical response of the Ag-GO nanohybrid materials obtained at higher ablation times can be explored in future advancements of this work, and the tunability in the unique structural and morphological features of such samples pave the way to explore possibilities with its synergistic effects for getting far better OL properties. The results highlight the potential utility of as-prepared colloidal Ag-GO hybrid nanostructures in photonic applications such as optical shutters, laser safety devices, etc.

## 4. Conclusions

In conclusion, the metallic Ag NPs have been effectively anchored on GO nanosheets via pulsed laser ablation of Ag target in an aqueous suspension of GO. The formation of surfactant-free colloidal Ag-GO hybrid materials of nanometer dimensions was also confirmed from absorption and Raman spectroscopy and TEM studies. The HRTEM images indicate the d-spacing and clear lattice fringes. The laser-synthesized nanohybrids exhibit excellent antimicrobial properties against *S. aureus* and *E. coli*. The enhancement of antibacterial activity with the addition of the Ag NPs on the surface of GO nanolayers may recommend tailoring of colloidal nanohybrid materials to be accepted for various clinical practices, including disinfection and antibacterial activities. Additionally, the drastic improvement in the NLA of the prepared nanohybrids could be because of the interplay between GO and the intermediate energy states of Ag NPs. The photon-induced charge carriers in the Ag-GO conduction bands or the introduction of so many defect states in the hybrid structure might also give rise to FCA and ESA and thus the enrichment in the NLA. Since the unification of GO and Ag NPs is often perused for the accomplishment of colloidal hybrid nanomaterials with optical, photocatalytic, and photoelectric applications. The present findings are beneficial for monitoring the design and development of similar nanomaterials, as well as for the evolution of nanohybrids with distinctive optical properties using pulsed laser ablation.

## Figures and Tables

**Table 1:** Inhibition zone diameters of *E. coli* and *S. aureus* bacteria treated with Ag-GO@355 nm and Ag-GO@532 nm samples.

Sample Code	Zone of Inhibition (in cm)	
	*E. coli*	*S. aureus*
GO	0.3	0.5
Ag	0.7	0.7
Ag-GO@355 nm	1.2	1
Ag-GO@532 nm	1.7	1.4

**Table 2:** Inhibition zone diameters of *E. coli* and *S. aureus* bacteria treated with Ag-GO@532 nm samples synthesized at different time scales.

Notations in the Images [Figure 5c,d]	Sample Code	Zone of Inhibition (in cm)	
		*E. coli*	*S. aureus*
1	GO	0.3	0.5
2	Ag-GO@532 nm@15 min	1.7	1.4
3	Ag-GO@532 nm@18 min	2	2
4	Ag-GO@532 nm@21 min	2.7	2.2
5	Ag-GO@532 nm@24 min	2.9	2.5
6	Ag-GO@532 nm@27 min	3	2.8

**Table 3:** Values of nonlinear absorption coefficient (*β_eff_*) and saturation intensity (*I_Sat_*) for excitations in the nanosecond regime.

Sample	Energy (µJ)	*β_eff_* (× 10^−10^ mW^−1^)	*I_sat_* (× 10^10^ Wm^−2^)
**Ag NPs**	40	0.19	420
**GO**	40	0.75	150
**Ag-GO@ 355 nm**	40	0.78	119
**Ag-GO@ 532 nm**	40	1.5	29
